# Parenting — a paradigm for investigating the neural circuit basis of behavior

**DOI:** 10.1016/j.conb.2019.11.011

**Published:** 2020-02

**Authors:** Johannes Kohl

**Affiliations:** The Francis Crick Institute, 1 Midland Rd., London NW1 1AT, UK

## Abstract

Parenting is essential for survival and wellbeing in many species. Since it can be performed with little prior experience and entails considerable sacrifices without immediate benefits for the caregiver, this behavior is likely orchestrated by evolutionarily shaped, hard-wired neural circuits. At the same time, experience, environmental factors and internal state also make parenting highly malleable. These characteristics have made parenting an attractive paradigm for linking complex, naturalistic behavior to its underlying neural mechanisms. Recent work — based on the identification of critical neuronal populations and improved tools for dissecting neural circuits — has uncovered novel functional principles and challenged simplistic models of parenting control. A better understanding of the neural basis of parenting will provide crucial clues to how complex behaviors are organized at the level of cells, circuits and computations. Here I review recent progress, discuss emerging functional principles of parental circuits, and outline future opportunities and challenges.

## Highlights

• Molecularly defined nodes have been identified in parental circuits.
• A functional circuit logic of parental behavior is emerging.
• Parenting relies on largely similar circuitry in males and females.
• Infant-directed aggression is controlled by dedicated circuits.

**Current Opinion in Neurobiology** 2020, **60**:84–91

This review comes from a themed issue on **Neurobiology of behavior**

Edited by **Richard Mooney** and **Michael Brecht**

For a complete overview see the Issue and the Editorial

Available online 9th December 2019

**https://doi.org/10.1016/j.conb.2019.11.011**

0959-4388/Crown Copyright © 2019 Published by Elsevier Ltd. This is an open access article under the CC BY license (http://creativecommons.org/licenses/by/4.0/).

## Introduction

Building on many decades of research in mammalian model systems, major progress has recently been made in understanding the circuit basis of parental behavior in laboratory mice (*Mus musculus*). Mice are ideally suited to this purpose since they exhibit robust parental care and are genetically tractable. Moreover, powerful tools for circuit mapping and interrogation are available for this species. Neuronal populations crucial for parenting have now been identified and a *functional circuit diagram* underlying parental behavior is taking shape. While these advances have refined previous models and revealed novel principles, they have also uncovered a considerable complexity. Key questions — such as whether parenting relies on dedicated circuits or, rather, generic circuits for social behavior — remain unaddressed. Here I review recent progress, present an emerging circuit logic of parental behavior and outline future challenges. I will first focus on neuronal populations critical for parental behavior before describing an updated functional circuit diagram for parenting. Next, I will discuss the negative regulation of parenting, with novel evidence suggesting that infant-directed aggression is an active process governed by dedicated circuits. Finally, I will outline potential avenues towards a systems-level interrogation of parental behavior.

### Neuronal populations critical for parenting

Although strongly modified by experience and physiological state, parenting is an instinctive behavior that can be displayed without any prior experience [1]. For instance, a strain-dependent proportion of virgin female laboratory mice for instance will display spontaneous parental behavior upon first encountering pups, comprising essentially all components of female parental behavior (grooming, licking, crouching, nest building), with the exception of nursing [1]. Similarly, virgin males, in which vomeronasal sensing is abolished, show paternal behavior instead of pup-directed aggression [2,3]. These observations suggest that functional parental circuits are present in adults of both sexes, and that genetic programs strongly contribute to the formation of such circuits. As a consequence, nodes in these circuits are likely composed of defined neuronal populations.

**Figure 1:**
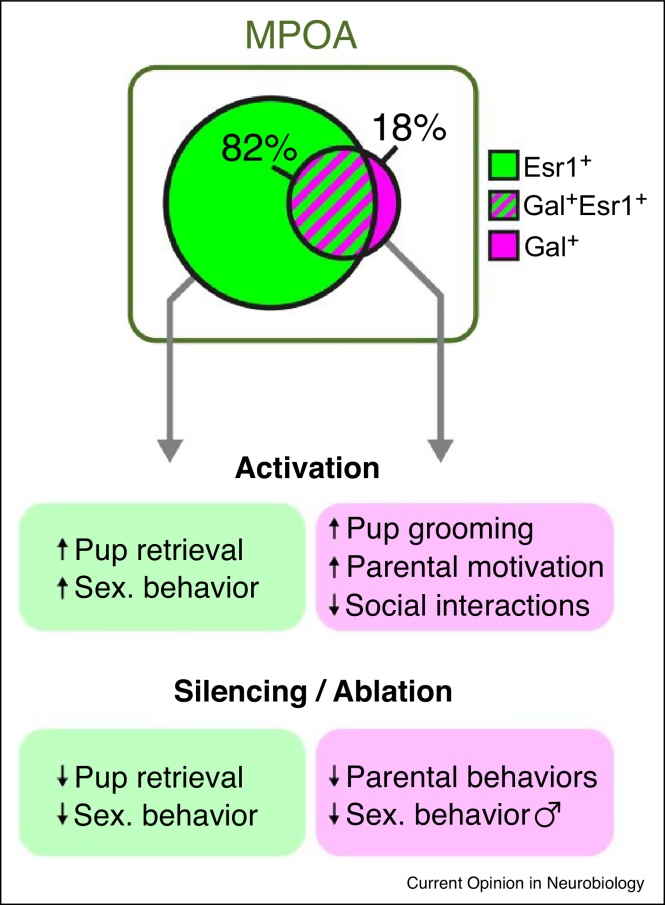
Two parenting-relevant neuronal populations in the MPOA. Distribution of, and overlap between, MPOA^Gal^ and MPOA^Esr1^ neurons are shown, as well as the behavioral consequences of manipulating each population. Note that about 90% of MPOA^Gal^ neurons, and more than 80% of MPOA^Esr1^ neurons, are GABAergic [2,12^••^]. For further details see text. Data from refs. [2,10^••^,12^••^] and JK *(unpublished)*. Unless specified, manipulations affect behavior in both sexes.

The use of cell type-specific manipulations has considerably advanced our understanding of how parenting as a complex social behavior is organized at the neural level. Most investigations have focussed on brain areas previously identified as critical for parenting by classic lesion studies, such as the medial preoptic area (MPOA) or the posterodorsal medial amygdala (MeApd) [4,5]. Within these areas, neuropeptides, neurotransmitters and receptors have typically been chosen as cellular markers — especially in the hypothalamus, which is composed of a rich set of distinct neuronal cell types [6,7,8^•^]. In addition, immediate early genes (IEGs, e.g. *c-fos*) are frequently used as indirect molecular readouts of neural activity to determine which neurons within such target areas are activated by a given behavior. These approaches have identified parenting-relevant neuronal populations and paved the way for dissecting the circuits within which these neurons function [9^••^,10^••^,11^•^,12^••^]. An initial study from Wu *et al.* reported that MPOA neurons expressing the neuropeptide Galanin (MPOA^Gal^ neurons), which comprise ∼20% of MPOA neurons, are crucial for parental behavior in both sexes (Figure 1) [2]. Two further studies found estrogen receptor α — expressing MPOA neurons (MPOA^Esr1^) to be critical for pup retrieval in females (Figure 1) [10^••^,12^••^]. Intriguingly, MPOA^Esr1^ neurons also strongly affect sexual behavior in males and females [12^••^].

These observations illustrate several important considerations when using genetic markers for circuit-level studies of behavior: (1) Genetic markers are necessarily imperfect, that is, not all neurons activated by, or involved in controlling, a given behavior, express a single marker. Conversely, not all marker-expressing neurons are involved in a given behavior. Neuropeptide expression can be associated with functional specialization (e.g. somatostatin-positive or parvalbumin-positive interneurons, oxytocinergic and vasopressinergic secretory neurons), but such populations are typically involved in narrowly described physiological functions. In contrast, circuits for complex behaviors are unlikely to be defined by single markers. Pragmatic considerations, for example, the availability of Cre mouse lines with restricted expression patterns, seem to underlie marker choice in some cases. (2) In cases where a marker is expressed by the majority of neurons within a brain area (e.g. >50% of MPOA neurons are Esr1-positive (Figure 1) [11^•^] and ∼70% of MeApd neurons are GABAergic [13]), the fact that the neurons in question express a marker might be largely irrelevant. Since individual brain areas participate in many behaviors and physiological functions, manipulation of a large fraction of neurons in an area would be expected to result in context-specific effects. This might explain why optogenetic activation of MPOA^Esr1^ neurons elicits context-dependent sexual-behavior or parental-behavior (Figure 1) [12^••^]. Another prediction is that manipulating variable fractions of a broad population (e.g. by tuning illumination levels in optogenetic experiments) would result in different phenotypes. In cases where the large majority of neurons within an area is manipulated, the conceptual advance over classic, non-cell type specific approaches is questionable. Screening for markers with high *enrichment ratios*, that is, controlling for relative frequency of marker-positive neurons within an area can address this limitation (see [2]). (3) Immediate early genes such as *c-fos* are slow (minutes-hours) and only provide an indirect readout of neural activity. Also, it remains incompletely understood which neuronal activity patterns result in their activation *in vivo* [14]. IEG-positive and marker-positive neurons thus only partially reflect parenting-relevant neural populations. These limitations also apply to other systems, such as Esr1-expressing neurons in the ventrolateral ventromedial nucleus of the hypothalamus (VMHvl^Esr1^), which have prominent roles in aggression [15] but also food intake, physical activity and thermogenesis [16,17].

Single-cell and spatial transcriptomics approaches now offer the opportunity to further define neuronal populations based on location, anatomical connectivity and gene expression profile [8^•^,^18^, ^19^, ^20^, ^21^, ^22^]. Several recent studies have used such approaches on hypothalamic populations [6,8^•^]. For instance, Moffitt *et al.* recently assembled a spatially resolved molecular atlas of the MPOA, identifying distinct MPOA^Gal^ subpopulations [8^•^]. In order to functionally exploit such refined molecular identities, better *genetic access* to such neuronal populations is required. At present, neurons characterized by expressing single marker genes are typically targeted using recombinase-expressing mouse lines. Only a handful of orthogonal recombinases (Cre, Flp, Dre, ΦC31, Vika) are currently available [23, 24, 25, 26]. Of those, Cre accounts for the vast majority and the generation of new lines is slow and expensive. Genetic intersections therefore remain challenging and impractical. Alternatively, conditional viral tools, especially adeno-associated viruses (AAVs), can be used. While their limited packaging capacity (∼4.7 kb) often precludes the incorporation of promoter fragments large enough to drive cell-type specific transgene expression (but see e.g. [27,28]), enhancer sequences have been shown to be suitable for this purpose [27,29]. Such approaches have the potential to give access to more specific, behaviorally relevant neuronal populations in the future.

### Circuit logic of parenting

Behaviors are encoded by dynamic activity patterns in brain-wide circuits. Although specific neuronal populations can neither be necessary nor sufficient for any given behavior [30], the identification of parenting-relevant neuronal populations has recently precipitated rapid advances in our understanding of how parenting is orchestrated at the circuit level [9^••^,12^••^,^31^,32^•^]. Lesion studies and pharmacological manipulations, primarily in female rats, have found many brain areas to be involved in parenting [1,9^••^,^33^,^34^]. Importantly, each of these areas is also critical for other social and non-social behaviors. Based on these seminal studies, a circuit model for parenting was proposed in which two opposing pathways mediate the activation and inhibition of parenting, respectively [1]. Chemosensory pup stimuli are integrated by the MeA, which exerts a negative effect on parenting by directly inhibiting the MPOA and by activating a ‘central aversion network’, encompassing the (ventral) lateral septum (LS), anterior hypothalamic nucleus (AH), VMH, dorsal premammillary nucleus (PMd) and periaqueductal gray (PAG). In contrast, the MPOA and adjacent ventral bed nucleus of the stria terminalis (vBNST) promote parenting, controlling its distinct components via dedicated downstream projections [1].

**Figure 2:**
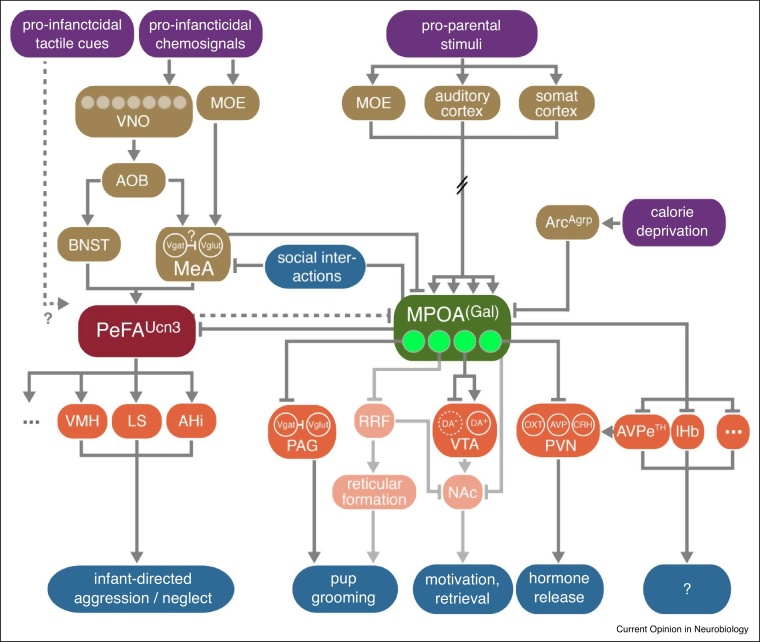
Emerging circuit logic underlying parental behavior. This functional circuit diagram is based on pharmacological and lesion- studies in virgin female rats [1], and extended by recent findings (see text, refs. [9^••^,10^••^,11^•^,12^••^,32^•^,^37^,40^••^,^42^,53^••^]). Arc^Agrp^ neurons, which sense caloric need and mediate feeding behavior, project to a subset of MPOA neurons [11^•^]. Optogenetic stimulation of this projection decreases maternal nestbuilding [11^•^]. Tyrosin hydroxylase-expressing neurons in the anteroventral periventricular nucleus (AVPe^TH^ neurons) are critical for parental behavior in females [42]. These neurons form monosynaptic connections with oxytocin-expressing neurons in the paraventricular hypothalamic nucleus, thereby influencing oxytocin release [42]. Abbreviations: AHI, amygdalohippocampal area; AOB, accessory olfactory bulb; AVPe, anteroventral periventricular nucleus; BNST, bed nucleus of the stria terminalis; LC, locus coeruleus; LS, lateral septum; lHb, lateral habenula; MeA, medial amygdala; NAc, nucleus accumbens; PVN, periventricular hypothalamic nucleus; PVT, periventricular thalamic nucleus; RRF, retrorubral field; SNpc, substantia nigra pars compacta; somat ctx, somatosensory cortex; SON, supraoptic nucleus; Vglut, vesicular glutamate transporter; Vgat, vesicular GABA transporter; VMH, ventromedial hypothalamus; VTA, ventral tegmental area.

Recent work in mice has begun to develop this region-level *wiring diagram* (lacking cellular identity and signs of synaptic connections) into a *functional circuit diagram* (Figure 2), starting from genetically defined populations such as MPOA^Gal^ neurons. Conditional retrograde trans-synaptic and anterograde viral tracers have been used to anatomically delineate elements of the circuit in which MPOA^Gal^ neurons are embedded [9^••^]. These neurons project to, and receive inputs from, more than 20 brain areas in a circuit exhibiting extensive reciprocity [9^••^]. Importantly, MPOA^Gal^ neurons form projection-defined subpopulations, each receiving inputs from essentially all input areas (Figure 2) [9^••^]. The parallel organization of MPOA^Gal^ projections is similar to what has been described for agouti-related peptide-expressing neurons in the arcuate nucleus (Arc^Agrp^ neurons) [35], but contrasts with, for example, VMH^Esr1^ or PeFA^Ucn3^ neurons (see ‘Negative regulation of parenting’), which predominantly send out branched projections [36,37]. Corresponding with this segregated organization, different MPOA^Gal^ pools are active during different episodes of parenting, and control distinct motor, motivational and hormonal aspects of parenting (Figure 2) [9^••^]. For instance, projections to the periaqueductal gray (PAG) are critical for pup grooming, which recapitulates the effect of optogenetically activating the entire MPOA^Gal^ population [2]. In contrast, MPOA^Gal^ projections to the ventral tegmental area (VTA) seem to control the motivation to interact with pups [9^••^]. In a separate study, Fang *et al.* reported that stimulating VTA-projecting MPOA^Esr1^ neurons elicits pup retrieval to the nest [10^••^], identical to what is observed when *all* Esr1-expressing or GABA-expressing MPOA neurons are activated [11^•^,12^••^]. VTA-mediated pup retrieval might be a consequence of acutely increased parental motivation (stimulation of MPOA^Esr1^ neurons also elicits retrieval of rubber pups [12^••^]), but further experimental evidence is needed to address the role of this projection. While it remains to be shown whether these projection-defined MPOA subpopulations have separable genetic identities (see e.g. [8^•^]), these results indicate that discrete components of a complex behavior can be isolated at the circuit level.

In addition to such efforts to trace parenting-relevant circuits in an *inside-out* manner, i.e. starting from neuronal populations deep in the brain, another possibility is to define parental circuits in an *outside-in* manner, starting from the sensory periphery. Such efforts have encountered both methodological and conceptual hurdles. One technical challenge is the absence of suitable reagents for anterograde trans-synaptic circuit tracing, although progress has recently been made in this regard [38]. Other limitations are of a conceptual nature: Because of their presumed ability to ‘trigger’ instinctive behaviors, pheromonal cues have long been proposed to be processed along dedicated, stimulus-specific neural circuits from the sensory periphery into the brain (*labeled lines*) [39]. Pup-emitted pheromones are thought to promote pup-directed aggression, since ablating vomeronasal organ (VNO) function elicits paternal behavior in otherwise infanticidal virgin males [2,3]. The identification of pup-specific vomeronasal receptors (VRs) might therefore constitute entry points into labeled line circuits into the brain. However, a recent study found that neither pup-sensitive vomeronasal receptors nor associated cues are pup-specific [40^••^]. Instead, such receptors are also tuned to adult chemosensory signals, and pup recognition relies on a combination of physical and chemical traits (see ‘Negative regulation of parenting’) [40^••^]. These findings thus call into question the existence of labeled lines for pheromone-triggered behavior [39,41], and therefore the possibility of an *outside-in* identification of parental circuits.

In summary, considerable progress has been made in uncovering the functional circuit architecture underlying parental behavior. Key emerging principles are that these circuits are enormously complex, overall remarkably similar between the sexes (but see [32^•^,^42^]), and that specific aspects of parenting can indeed be assigned to discrete circuit elements [9^••^,^43^]. It will be interesting to investigate how this circuitry interacts with neural systems controlling other instinctive behaviors (or whether they largely overlap), how information is processed between successive circuit nodes and how experience and physiological states affect their function.

### Negative regulation of parenting

Under certain physiological and environmental conditions, animals neglect or attack young conspecifics. Males in some species kill unfamiliar infants to gain reproductive advantage [44, 45, 46] and females neglect or attack their young during stressful circumstances such as food shortage or threat of predation [47]. In laboratory mouse strains, which are a product of artificial selection for a variety of physiological and behavioral traits [45,47,48], aggressive behavior is less pronounced. Infant-directed aggression has predominantly been studied in males, which undergo a switch from infanticide to paternal care after mating [2,3,49,50]. This striking phenomenon seems to be synchronized with the female’s gestation time to ensure paternity. Does infanticide simply result from downregulating parental circuits, or is it rather orchestrated by dedicated circuits? Several lines of evidence now indicate that it is a combination of both, as I will outline below.

In the periphery, detection of pup-emitted chemosensory signals is crucial for male infanticide, since this behavior is abolished by surgical or genetic ablation of VNO function [2,3]. Two recent studies have identified relevant cues and neurons involved in their detection: Trouillet *et al.* found that conditional ablation of the G-protein subunit Gαi2 — expressed in a subclass of VNO neurons — reduces infant-directed aggression. In a complementary study, Isogai *et al.* systematically screened for VNO neurons (which each typically express a single VR) activated by pup cues. They identified a repertoire of 7 VRs, knock-out of two of which, Vmn2r65 and Vmn2r88 (both Gαi2-negative), significantly decreased pup-directed aggression in virgin males [40^••^]. Together, these results suggest that several VRs (and, correspondingly, VNO neuron types) contribute to the detection of infant cues. Surprisingly, however, these VRs are also activated by adult cues, and pup recognition requires a combination of chemical and tactile cues [40^••^]. Furthermore, the chemical stimuli detected by Vmn2r65 and Vmn2r88 are rather unexpected: submandibular gland protein C, expressed in salivary glands of pups and adult females, and hemoglobins, which are ubiquitously found in social environments, especially after parturition [40^••^]. These results indicate that VNO cues emitted by infants are ambiguous, and that adults use multisensory information for pup recognition.

How are pro-infanticidal stimuli processed deeper in the brain? Vomeronasal information is relayed to the MeA via the accessory olfactory bulb (AOB) before reaching hypothalamic areas, such as the BNST or MPOA (Figure 2) [51]. Chemosensory signals from both VNO and the main olfactory system are presumably integrated by MeA neurons [52], but it remains unclear where and how these signals interact with haptic and other types of sensory information to form pup representations (Figure 2). Intriguingly, ablation of Gαi2 suppresses infanticide, but enhances male-male aggression [53^••^]. Together with the observation that the MeA neurons activated during infanticide are different from those involved in male-male aggression [53^••^], this suggests that these aggressive behaviors are controlled by different circuit mechanisms. MeA lesions facilitate parental behavior in females, and activation of GABAergic MeA neurons mirrors this effect [32^•^,^54^]. The effects of MeA lesions on pup-directed behavior in males are unclear, but Chen *et al.* recently reported that optogenetic activation of GABAergic MeA neurons can result in either parental behavior or infanticide, depending on illumination strength [32^•^]. Since the large majority of MeA neurons are GABAergic [55], these effects might be the consequence of activating neuronal subpopulations with distinct roles (see ‘Neuronal populations critical for parenting’). Located further along the pheromone processing pathway, lesions to the rhomboid nucleus of the BNST (BSTrh) were shown to suppress infanticidal behavior [56^•^], and functional maturation of BSTrh inputs during adolescence has been hypothesized to underlie the change from parental to infanticidal behavior [57^•^]. In order to identify additional infanticide-relevant regions, a recent study used brain-wide IEG mapping, uncovering a marked upregulation of c-Fos in the caudal hypothalamus after pup-directed aggression [58]. Autry *et al.* subsequently investigated this region in greater detail and found that Urocortin 3-expressing neurons in the perifornical area (PeFA^Ucn3^ neurons) are activated during pup-directed, but not male-male, aggression in both sexes [37]. While silencing of PeFA^Ucn3^ neuronal activity in virgin males blocks infanticide, activation of these neurons elicits infant-directed neglect in virgin females [37]. Intriguingly, PeFA^Ucn3^ neurons receive direct inputs from (almost exclusively inhibitory) MPOA^Gal^ neurons [2], suggesting that infanticide-promoting circuits might be actively suppressed in parental animals.

Altogether, these observations indicate that (1) infant-directed aggression relies on dedicated circuits which are likely distinct from those mediating male-male aggression, (2) these circuits directly interact with parental circuits — potentially in a mutually inhibitory fashion, and (3) similar neural mechanisms control infant-directed aggression in males and females. It will be exciting to further dissect the circuit mechanisms underlying infant-directed aggression, to investigate how stress promotes this behavior in females, and to address which plasticity mechanisms govern the switch from infanticide to parenting in males.

### Towards a systems-level investigation of parental behavior

A key insight from recent studies is that parenting, as well as other instinctive behaviors, rely on highly complex, unexpectedly malleable, and potentially overlapping circuits [9^••^,^35^,^59^,^60^]. It remains unclear whether parental behavior is controlled by parenting-specific circuits or rather by general-purpose social behavior circuits that are state-specifically and/or context-specifically engaged. Distinguishing between these scenarios will require the use of systems neuroscience approaches and the integration of anatomical, functional and behavioral data.

First, single cell and spatial transcriptomics approaches have the potential to identify novel genetic entry points into parenting-relevant neuronal populations, and to uncover plasticity mechanisms within these populations. For instance, Moffitt *et al.* recently used a massively multiplexed *in situ* hybridization pipeline (MERFISH) to create a cell atlas of the preoptic area, defining novel cell types and subdividing MPOA^Gal^ neurons into ten transcriptionally and spatially distinct clusters [8^•^]. Second, refined anatomical approaches will help uncover further motifs in parental circuits, thereby guiding future functional investigations. Improved viral vectors now enable more specific, efficient and permanent access to defined neurons and circuits [61, 62, 63, 64, 65]. However, viral tracing approaches typically visualize connectivity between hundreds to thousands of neurons, thereby obscuring cellular-level anatomical diversity. Individual neurons can be reconstructed by serial two-photon tomography after sparse neuronal labeling, which revealed strikingly complex morphologies and brain-wide projection patterns [66,67]. However, this approach is is highly time-consuming, resource-intensive and laborious. High-throughput, sequencing-based strategies, such as MapSeq [68] are expected to give complementary insights into the organizational principles of parenting-relevant circuits. Third, rather than investigating these circuits one node at a time, addressing dynamic information processing at brain-wide scales will be necessary to understand the neural computations underlying parenting and other instinctive behaviors. High-density recordings from thousands of individually resolved neurons across the brain, will be instrumental for tracking information flow within circuits [69, 70, 71, 72]. Lastly, deep learning approaches now allow for automated, marker-less tracking of animals under varying experimental conditions, thereby greatly reducing the time required to analyze behavioral video recordings [73, 74, 75]. These methods have facilitated behavioral *tracking*, but behavioral *classification* remains challenging (e.g. pup grooming versus chemoinvestigation), especially for social interactions involving several subjects. Further improvements to these algorithms, assay-specific behavioral classifiers, and optimization of experimental conditions will without doubt result in increasingly automated behavioral quantification.

Fully leveraging these methodologies will put us in a position to address key questions in neuroscience, such as the degree of plasticity within neural circuits thought to be hardwired, how robustness and plasticity are balanced in such systems, and whether circuits for different behaviors are separate or highly overlapping. Thus, insights into the neural mechanisms underlying parental behavior have the potential to broadly contribute to our general understanding of how evolutionarily sculpted circuits control instinctive behaviors.

## Conflict of interest statement

Nothing declared.

## References and recommended reading

Papers of particular interest, published within the period of review, have been highlighted as:

• of special interest
•• of outstanding interest
